# Effect of Ionic Correlations on the Surface Forces in Thin Liquid Films: Influence of Multivalent Coions and Extended Theory

**DOI:** 10.3390/ma9030145

**Published:** 2016-03-01

**Authors:** Krassimir D. Danov, Elka S. Basheva, Peter A. Kralchevsky

**Affiliations:** Department of Chemical and Pharmaceutical Engineering, Faculty of Chemistry and Pharmacy, Sofia University, Sofia 1164, Bulgaria; kd@lcpe.uni-sofia.bg (K.D.D.); eb@lcpe.uni-sofia.bg (E.S.B.)

**Keywords:** liquid films, disjoining pressure, surface forces, multivalent ions, asymmetric electrolytes, ion correlations, Debye length, DLVO theory

## Abstract

Experimental data for the disjoining pressure of foam films stabilized by anionic surfactant in the presence of 1:1, 1:2, 1:3, and 2:2 electrolytes: NaCl, Na_2_SO_4_, Na_3_Citrate, and MgSO_4_ are reported. The disjoining pressure predicted by the Derjaguin-Landau-Verwey-Overbeek (DLVO) theory coincides with the experimental data in the case of a 1:1 electrolyte, but it is considerably greater than the measured pressure in all other cases. The theory is extended to account for the effects of ionic correlations and finite ionic radii. Original analytical expressions are derived for the local activity coefficient, electrostatic disjoining pressure, and asymptotic screening parameter. With the same parameter of counterion binding as for a 1:1 electrolyte, the curves predicted by the extended theory are in perfect agreement with the experimental data for 1:2 and 1:3 electrolytes. In comparison with the DLVO theory, the effect of ionic correlations leads to more effective screening of electrostatic interactions, and lower electric potential and counterion concentrations in the film’s midplane, resulting in lower disjoining pressure, as experimentally observed. The developed theory is applicable to both multivalent coions and multivalent counterions. Its application could remove some discrepancies between theory and experiment observed in studies with liquid films from electrolyte solutions.

## 1. Introduction

The Derjaguin-Landau-Verwey-Overbeek (DLVO) theory [1,2] of surface forces in thin liquid films provides a quantitative description of the behavior of colloidal dispersions. Later, additional effects called non-DLVO forces have been taken into account [3]. In fact, one of the basic equations for the electrostatic component of disjoining pressure of a symmetric plane-parallel liquid film, Π_el_, was proposed earlier by Langmuir [4]:

(1.1) $$\Pi_{\text{el}}=p(x_{m})-p_{\infty }=kT\;[c_{1}(x_{m})+c_{2}(x_{m})-(c_{1\infty }+c_{2\infty })]$$

Here, *k* is the Boltzmann constant; *T* is the absolute temperature; the *x*-axis is directed perpendicular to the film surfaces; *x*_m_ is the coordinate of the film’s midplane; *p*(*x*_m_) is the osmotic pressure in the midplane; and *c*_1_(*x*_m_) and *c*_2_(*x*_m_) are the concentrations of coions and counterions therein; whereas *p*_∞_, *c*_1∞_, and *c*_2∞_ are the values of the respective quantities in the bulk of solution. Equation (1.1) expresses Π_el_ as an excess osmotic pressure of the ions in the middle of the film, where the electric field is equal to zero. The Debye-Hückel theory yields the following expression for the osmotic pressure of solutions of strong electrolytes [5,6]:

(1.2) $$p(x)=kT\;[c_{1}(x)+c_{2}(x)]-\frac{kT}{24\pi }{\left\{\frac{e^{2}}{\varepsilon_{0}\varepsilon \;kT}[z_{1}^{2}c_{1}(x)+z_{2}^{2}c_{2}(x)]\right\}}^{3/2}$$

where *ε*_0_ is the dielectric permittivity of vacuum; *ε* is the relative dielectric constant; *e* is the charge of positron; and *z*_1_ and *z*_2_ are the valences of the respective ions. The last term in Equation (1.2), which takes into account the effect of ion-ion correlations, gives an essential contribution to the value of *p* even for moderately high electrolyte concentrations, but this effect is not included in the DLVO theory [7]. Indeed, only the contribution of the first term in the right-hand side of Equation (1.2) has been taken into account in Equation (1.1). Nevertheless, the ionic correlations are expected to influence the electrostatic interactions in the thin liquid films and colloidal dispersions, especially in the presence of multivalent ions.

In the case of single electric double layer, Outhwaite and Bhuiyan [8] proposed an approach based on a modified Poisson-Boltzmann equation, which takes into account the contribution of ionic correlations to the double-layer potential and the coion and counterion profiles. In the case of two overlapping double layers (thin liquid film), Attard *et al.* [9] proposed an analytical approach, which extends the DLVO theory based on the Poisson-Boltzmann equation by including the effect of images and ionic correlations in the case of symmetric electrolytes. Ninham *et al.* [10,11] derived an analytical expression for the decay length of the asymptotic form of the double-layer force in mixed and asymmetric electrolytes, which is markedly different from the classical Debye length. The latter expression was verified experimentally by Pashley *et al.* [12] by using the method of colloidal-probe atomic force microscopy (CP-AFM). These authors concluded that the various proposed corrections to the electrostatic screening lengths in solutions of multivalent electrolytes are either invalid or beyond the limits of measurement of the used technique. It was found that surface force profiles measured by CP-AFM in solutions with monovalent counterions and multivalent coions were accurately described by the DLVO theory [13]. However, differences between the predictions of the Monte Carlo simulations and the DLVO theory have been reported in several studies [14,15,16,17] for solutions of asymmetric electrolytes.

In the case of 2:1 electrolytes, the divalent counterions adsorb strongly on the film surfaces and decrease the double-layer repulsion [18]. In the case of multivalent counterions, even charge reversal was observed [19,20,21]. The charge reversal is related to the effect of counterion adsorption, termed also counterion binding or condensation. Besteman *et al.* [22,23] showed that, in the presence of sufficiently high concentrations of multivalent salt, two originally oppositely charged surfaces could repel each other. Later, Monte Carlo simulations showed that this repulsion appears to be a consequence of ion-ion correlations in the film [24]; see also [25]. By theoretical analysis, strong evidence was found that ion–ion correlations can be the origin of charge inversion in the electric double layer near the interface between mercury and an aqueous MgSO_4_ solution [26].

At present, the results about the importance of the effect of ionic correlations for the electrostatic double-layer interactions in thin liquid films appear to be controversial. In part, this is due to the fact that in many cases the correlation effect could be incorporated in an effective surface charge (or potential) [9], which can be determined as an adjustable parameter, thus obtaining an apparent agreement with the DLVO theory. The binding energies of counterions of various valences, which are frequently unknown, lead to the appearance of additional adjustable parameters. To overcome this problem, in the present study we measured the disjoining pressure of free thin liquid films stabilized by the anionic surfactant sodium dodecyl sulfate (SDS). Disjoining pressure isotherms were measured in the presence of 1:1; 1:2, and 1:3 electrolytes, *viz.* NaCl, Na_2_SO_4_, and Na_3_Citrate. In other words, the counterion, Na^+^, is the same, whereas the valences of the coions are different. It is very important that the binding energy of the Na^+^ ion to the sulfate headgroup of the SDS molecule, characterized by the Stern constant *K*_St,2_, is known from surface-tension studies [27,28,29,30] and has not been used as an adjustable parameter in the present study. In such a case, the effect of the ionic correlations on the surface force (if any) should be detected as a deviation from the predictions of the DLVO theory in regime of charge regulation, characterized by the same counterion binding constant, *K*_St,2_, for the 1:1, 1:2, and 1:3 electrolytes. Disjoining pressure isotherm has been measured also in the presence of MgSO_4_ to compare the ionic-correlation effects in the presence of 1:1 and 2:2 electrolytes.

Section 2 describes the used materials and methods. In Section 3, the DLVO theory is extended to take into account the effect of ionic correlations, henceforth referred to as DLVO-IC for brevity. In Section 4, the experimental data are compared with the extended theory; the effect of ionic correlations is identified and interpreted in terms of ion concentrations and electrostatic potential inside the film. In Section 5, an analytical expression is derived for the asymptotic decay length of double-layer interactions affected by the ionic correlations. In Appendix A, a general expression for the local activity coefficient in electric double layers is derived, which is used in the DLVO-IC theory. We should mention in advance that a considerable effect of ionic correlations was detected in the cases of 1:2, 1:3 and 2:2 electrolytes, and that DLVO-IC accurately describes the experimental disjoining pressure isotherms.

## 2. Materials, Methods and Experimental Results

The chemicals used were: sodium dodecyl sulfate (SDS) from Across Organics (Geel, Belgium); sodium chloride and sodium sulfate (NaCl and Na_2_SO_4_) from Merck (Kenilworth, NJ, USA); and sodium citrate and magnesium sulfate (Na_3_Citrate and MgSO_4_) from Sigma-Aldrich (Taufkirchen, Germany). All solutions were prepared with deionized water of specific resistivity 18.2 MΩ·cm (Elix purification system, Millipore, Molsheim, France). The natural pH of the Na_3_Citrate solutions was 7.0 ± 0.1, which guarantees that the citrate anion is present in its trivalent form. In all experiments, the concentration of SDS was 1 mM, whereas the ionic strength due to the added electrolyte was 30 mM. The last value corresponds to 30 mM NaCl, 10 mM Na_2_SO_4_, and 5 mM Na_3_Citrate. All experiments were carried out at a room temperature of 25 ± 1 °C.

**Figure 1:**
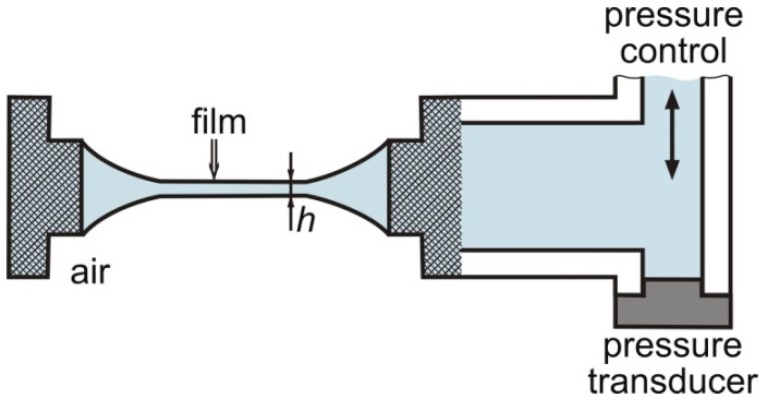
Sketch of the Mysels–Jones (MJ) cell [31], where a foam film is formed in the center of a hole in a porous plate, which is connected to a capillary filled with the surfactant solution and to a pressure transducer.

The dependence of disjoining pressure, Π, *versus* the film thickness, *h*_w_, was measured by means of a Mysels-Jones (MJ) experimental cell [31], which was further modified and termed thin film pressure balance [32,33]; see Figure 1. The film formed in the center of a cylindrical hole drilled in a porous-glass plate. The two film surfaces are forced against each other at pressures of up to 9000 Pa in our experiments. The film thickness was determined by an interferometric method from the intensity of the monochromatic light (*λ* = 546 nm) reflected from the film [32,34,35]. The value of film thickness was calculated assuming that the film is a uniform plane-parallel layer with refractive index of water, *n*_w_ = 1.33, and for this reason the determined optical thickness was called equivalent water thickness, *h*_w_. The reflected light was supplied to a photomultiplier and computer, and *h*_w_ was recorded continuously during the experiment. The applied pressure was measured electronically by a pressure transducer. A detailed description of the used version of the MJ cell can be found elsewhere [36].

**Figure 2:**
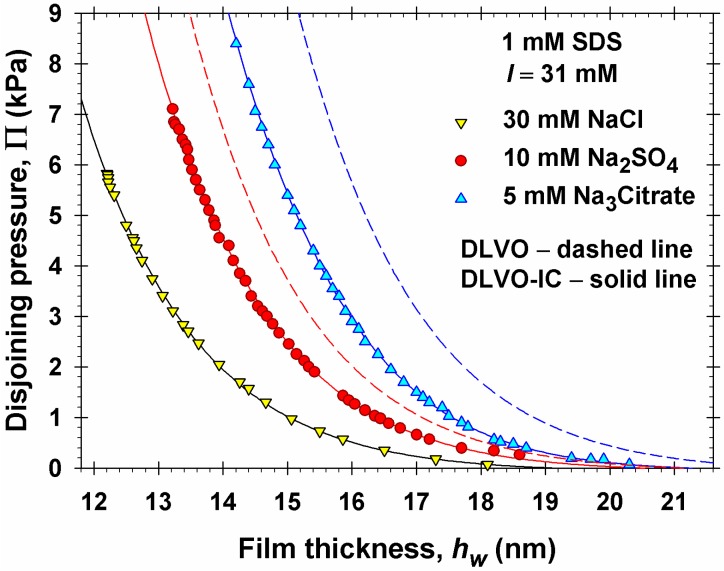
Dependences of disjoining pressure, Π, on the experimental film thickness, *h*_w_. The points are experimental disjoining pressure isotherms of thin foam films stabilized with 1 mM SDS in the presence of three different salts, NaCl, Na_2_SO_4_, and Na_3_Citrate, at the same total ionic strength, *I* = 31 mM. The dashed and solid lines are drawn using the conventional DLVO theory and the DLVO theory with ionic correlations (DLVO-IC). In the case of NaCl, the predictions of DLVO and DLVO-IC coincide.

During the experiment, the applied pressure was increased in small steps, of 40 Pa for Π < 3000 Pa, and of 100 Pa for Π > 3000 Pa. After each pressure increase, the computer’s indications for the film thickness were observed (from 5 to 30 min) until an equilibrium thickness was established, which was recorded together with the respective value of pressure. After that, the pressure was increased again to obtain the next experimental point. For each of the three electrolytes, NaCl, Na_2_SO_4_ and Na_3_Citrate, the experimental Π(*h*_w_) dependence was measured in four independent runs. The experimental points shown in Figure 2 are average values from these four experiments. The error of the mean value of *h*_w_ is ±0.4 nm for Π < 1 kPa; ±0.3 nm for 1 ≤ Π ≤ 4 (kPa); and ±0.15 nm for Π > 4 kPa.

Figure 2 shows the obtained experimental Π(*h*_w_) isotherms. As already mentioned, in all experiments the total ionic strength,

(2.1) $$I=\frac{1}{2}\sum_{i=1}^{n}z_{i}^{2}c_{i\infty }$$

was the same; *I* = 31 mM: 1 mM from the SDS and 30 mM from the added electrolyte; *z_i_* are the valences of the ions; and *c_i_*_∞_ are their bulk concentrations. Despite the fixed *I*, the Π(*h*_w_) isotherms measured in the presence of 1:1, 1:2, and 1:3 electrolytes differ essentially from each other: Π increases with the rise of coion valence. The predictions of the conventional DLVO theory coincide with the experimental Π(*h*_w_) curve only in the case of NaCl (computational details can be found below). In contrast, for the 1:2 and 1:3 electrolytes, the Π calculated by DLVO theory is considerably greater than the experimental Π. As demonstrated below, this discrepancy can be overcome if the DLVO theory is extended to account for the effect of ionic correlations. In the presence of asymmetric electrolytes, the ionic correlation effect leads to a stronger screening of the repulsive electrostatic interactions and to lower values of disjoining pressure, as experimentally observed (Figure 2). The effect is stronger for a 1:3 electrolyte. For a 1:2 electrolyte the effect is weaker, but noticeable, similarly to the difference between the Monte Carlo data and the DLVO curve in [14]. The greater reduction of the electrostatic repulsion with the rise of coion valence found in the present study is in qualitative agreement with the inverse Schulze-Hardy rule established in [37].

## 3. Generalized Theory of Electrostatic Surface Forces in Thin Liquid Films

### 3.1. Expression for Disjoining Pressure Extended to Account for Ionic Correlations

**Figure 3:**
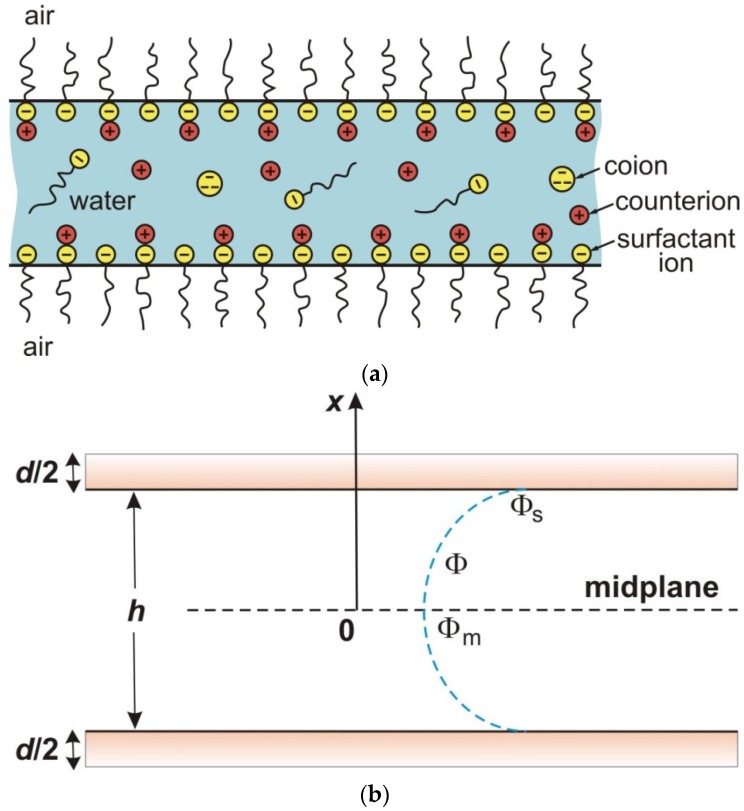
(**a**) Sketch of a foam film, like those studied in our experiments, with surfactant ions, counterions, and coions; (**b**) simplified scheme of a symmetric plane-parallel liquid film of inner thickness *h*, together with the distribution of electric potential Φ; Φ_s_ and Φ_m_ are the values of Φ at the film surface and midplane; *d* is the total thickness of the two adsorption layers.

Let us consider a free liquid film formed from a solution of surfactant and electrolyte; see Figure 3a. The film surfaces are electrically charged and the electric potential in the film, *ϕ*, obeys the Poisson equation:

(3.1) $$\varepsilon_{0}\varepsilon \frac{d^{2}\varphi }{dx^{2}}=-\rho_{b},\;\rho_{b}=\sum_{i=1}^{n}z_{i}ec_{i}$$

here, *x* is Cartesian coordinate normal to the film surfaces (Figure 3b); *ρ*_b_ is the local charge density in the electric double layer (EDL); *c_i_* = *c_i_*(*x*) are the local concentrations of the ions; and *n* is the number of ionic components. Under static conditions, the Navier-Stokes equation reduces to

(3.2) $$\frac{dp}{dx}=-\rho_{b}\frac{d\varphi }{dx}=\frac{d}{dx}[\frac{\varepsilon_{0}\varepsilon }{2}{\left(\frac{d\varphi }{dx}\right)}^{2}]$$

where *p* is the local osmotic pressure; and *ρ*_b_ has been substituted from the Poisson Equation (3.1). Equation (3.2), which expresses the local force balance in the fluid inside the film, implies:

(3.3) $$dp=-\rho_{b}d\varphi$$

Because the electrochemical potential, *μ_i_*, of the *i-*th ionic species is constant throughout the EDL, we obtain:

(3.4) $$0=c_{i}d\mu_{i}=kTc_{i}d\ln(\gamma_{i}c_{i})+z_{i}ec_{i}d\varphi$$

where *γ_i_* is the activity coefficient. Upon summation in Equation (3.4) over all ionic species, its last term becomes *ρ*_b_d*ϕ*, which in view of Equation (3.3) yields:

(3.5) $$dp=kT\sum_{i=1}^{n}c_{i}d\ln(\gamma_{i}c_{i})$$

The effect of ionic correlations on the local pressure, *p*, is incorporated in the activity coefficient, *γ_i_*. In Appendix A, using the Debye-Hückel theory extended to a nonuniform electrolyte solution (like an electric double layer), we have derived the following expression for *γ_i_*:

(3.6) $$\begin{array}{ll} \ln\gamma_{i}= & -\frac{z_{i}^{2}L_{B}\kappa }{{\left(\kappa \;r_{i}\right)}^{3}}[\kappa \;r_{i}(\frac{\kappa \;r_{i}}{2}-1)+\ln(1+\kappa \;r_{i})]\\ & -2\pi \frac{z_{i}^{2}L_{B}^{2}}{\kappa }\sum_{j=1}^{n}\frac{z_{j}^{2}c_{j}}{{\left(\kappa \;r_{j}\right)}^{2}}[\frac{2+\kappa \;r_{j}}{1+\kappa \;r_{j}}-\frac{2}{\kappa \;r_{j}}\ln(1+\kappa \;r_{j})] \end{array}$$

here, *r_i_* is the radius of the respective ion; *κ* is the *local* Debye screening parameter; and *L*_B_ is the Bjerrum length:

(3.7) $$\kappa^{2}\equiv 4\pi \;L_{B}\sum_{i=1}^{n}z_{i}^{2}c_{i}\;,\;L_{B}=\frac{e^{2}}{4\pi \varepsilon_{0}\varepsilon \;kT}$$

The integration of Equation (3.5), along with Equations (3.6) and (3.7), yields:

(3.8) $$p(c_{1},...,c_{n})=kT\sum_{i=1}^{n}c_{i}-\frac{1}{2}kT\kappa L_{B}\sum_{i=1}^{n}\frac{z_{i}^{2}c_{i}}{{\left(\kappa \;r_{i}\right)}^{2}}[\frac{2+\kappa \;r_{i}}{1+\kappa \;r_{i}}-\frac{2}{\kappa \;r_{i}}\ln(1+\kappa \;r_{i})]$$

By differentiation, one could verify that the expression for *p* in Equation (3.8) satisfies Equation (3.5). In the limit *κr_i_* → 0, Equation (3.8) reduces to Equation (1.2), generalized for the case of *n* ionic components. Moreover, the form of the last term in Equation (3.8) indicates that the correlation effect, related to the screening parameter, *κ*, is coupled with the effect of finite ionic radius, *r_i_*. The value of *p* in the bulk of solution can be calculated from Equations (3.7) and (3.8) with *c_i_* = *c_i_*_∞_:

(3.9) $$p_{\infty }=p(c_{1\infty },...,c_{n\infty })$$

where *c_i_*_∞_ (*i* = 1, …, *n*) are the respective bulk concentrations.

The constancy of the electrochemical potential in the electric double layer, leads to Boltzmann relations for the activities of the ions:

(3.10) $$\gamma_{i}c_{i}=\gamma_{i\infty }c_{i\infty }\exp(-z_{i}\Phi ),\;\Phi =\frac{e|\varphi |}{kT}$$

here, *i* = 1, …, *n*; *γ_i_* is the activity coefficient in the EDL given by Equation (3.6); *γ_i_*_∞_ is the activity coefficient in the bulk of the solution, which is to be calculated from Equation (3.6) with *c_i_* = *c_i_*_∞_; *ϕ* and Φ are the dimensional and dimensionless electric potential in the EDL; for convenience, Φ is defined as a nonnegative quantity.

The combination of Equations (3.8) and (3.10) defines *p* as a function of the electrostatic potential; *p* = *p*(Φ). The electrostatic component of disjoining pressure is defined by the expression [1,2,3,4,7]:

(3.11) $$\Pi_{\text{el}}=p(\Phi_{m})-p_{\infty }$$

where Φ_m_ and *p*(Φ_m_) are the values of Φ and *p*(Φ) in the film’s midplane (Figure 3b). Equation (3.11), along with Equations (3.6)–(3.10), represents an expression for the electrostatic disjoining pressure extended to account for the effect of ionic correlations. It has to be noted that Equation (3.8) should also contain an additive constant of integration (background pressure), which is canceled in Equation (3.11) and does not affect Π_el_.

### 3.2. Calculation of the Electrostatic Disjoining Pressure with Ionic Correlations

By integration of Equation (3.2), one derives:

(3.12) $$\frac{d\Phi }{dx}={\left\{\frac{8\pi L_{B}}{kT}[p(\Phi )-p(\Phi_{m})]\right\}}^{1/2}$$

A subsequent integration from the film midplane (*x* = 0) to the film surface (*x* = *h*/2) yields:

(3.13) $$\int_{\Phi_{m}}^{\Phi_{s}}{\left\{\frac{2\pi \;L_{B}^{3}}{kT}[p(\Phi )-p(\Phi_{m})]\right\}}^{-1/2}d\Phi =\frac{h}{L_{B}}$$

where *h* is the film thickness; and Φ_s_ is the dimensionless potential at the film surface. The boundary condition for the electric field at the film surface reads:

(3.14) $$\varepsilon_{0}\varepsilon \frac{d\varphi }{dx}=-\rho_{s}=-\sum_{i=1}^{n}z_{i}e\Gamma_{i}\mathrm{at}x=\frac{h}{2}$$

here, *ρ*_s_ is the surface charge density and Γ*_i_* is the adsorption of the respective ionic species, *i.e.*, the number of adsorbed ions per unit surface area. (We have Γ*_i_* = 0 if component *i* is not present at the film surface.) The elimination of the derivative of electric potential between Equations (3.12) and (3.14) yields:

(3.15) $$\frac{L_{B}^{3}}{kT}[p(\Phi_{s})-p(\Phi_{m})]=2\pi {\left(L_{B}^{2}\sum_{k=1}^{n}z_{k}\Gamma_{k}\right)}^{2}$$

As an example, our experimental system (see Section 2) contains three kinds of ions: component 1—surfactant ion (dodecyl sulfate anion); component 2—counterion (Na^+^), and component 3—coions (Cl^−^, SO_4_^2^^−^ or Citrate^3^^−^). In this case, Γ_1_ denotes the surfactant adsorption, Γ_2_ denotes the number of counterions bound per unit surface area, and Γ_3_ = 0, *i.e.*, the negatively charged coions are not expected to bind to the like charged interface. For our computations, it is convenient to present the surfactant adsorption isotherm [27] in the form:

(3.16) $$K_{1}\gamma_{1\infty }c_{1\infty }\exp(-\Phi_{s})=\;\frac{\Gamma_{1}}{\Gamma_{\infty }-\Gamma_{1}}\exp(\frac{\Gamma_{1}}{\Gamma_{\infty }-\Gamma_{1}}-\beta \frac{\Gamma_{1}}{\Gamma_{\infty }})-K_{1}\gamma_{1\infty }c_{1\infty }K_{\text{St},2}\gamma_{2\infty }c_{2\infty }$$

here, *K*_1_ is the surfactant adsorption constant; *K*_St,2_ is the Stern constant of Na^+^ binding to the surfactant headgroups in the adsorption layer; $\Gamma_{\infty }^{-1}$ is the excluded area per surfactant molecule in the adsorption layer; and *β* is a parameter that characterizes the attraction between the surfactant tails in the adsorption layer. All these parameters have been determined from the fits of surface tension isotherms at various salt concentrations [28]. Equation (3.16) corresponds to the van der Waals adsorption model, which turns out to be adequate for describing surfactant adsorption at a fluid interface [38]. In addition, the counterion binding can be described by the Stern isotherm [27]:

(3.17) $$\frac{\Gamma_{2}}{\Gamma_{1}}=\frac{K_{\text{St},2}\gamma_{2\infty }c_{2\infty }\exp(\Phi_{s})}{1+K_{\text{St},2}\gamma_{2\infty }c_{2\infty }\exp(\Phi_{s})}$$

The use of Equation (3.17) means that we are working in the regime of charge regulation, *i.e.*, in the regime of constant electrochemical potential, which is the real experimental situation. Indeed, Equation (3.17) is derived by setting the electrochemical potential of the counterions in the bulk equal to that of the adsorbed counterions (bound in the Stern layer), where the Langmuir adsorption isotherm has been used. Note that Equations (3.16) and (3.17) are thermodynamically compatible, *i.e.*, they satisfy the Euler condition for exact differential [27]:

(3.18) $$\frac{\partial \Gamma_{2}}{\partial \ln a_{1s}}=\frac{\partial \Gamma_{1}}{\partial \ln a_{2s}}$$

where *a_i_*_s_ = *γ_i_*_∞_*c_i_*_∞_exp(−*z_i_*Φ), *i* = 1, 2, are the subsurface activities of the ions.

The goal of the computational procedure is to obtain the theoretical disjoining pressure isotherm, Π_el_ = Π_el_(*h*), in a parametric form: Π_el_ = Π_el_(Φ_m_) and *h* = *h*(Φ_m_). The input parameters are the bulk concentrations of surfactant ions, *c*_1∞_; counterions, *c*_2∞_, and coions, *c*_3∞_, as well as a number of physical parameters given in the beginning of Section 4. In the computer program, all quantities are calculated with double precision. The computational procedure is as follows:

1. For a given value of Φ_m_, from Equations (3.6) and (3.10) we determine the respective values of the concentrations and activity coefficients, *c_i_* = *c_i_*_m_ and *γ_i_* = *γ_i_*_m_, by iterations. To start the iterations, first *c_i_* is calculated from Equation (3.10) with Φ = Φ_m_ and *γ_i_* = *γ_i_*_∞_. The obtained *c_i_* is substituted in Equations (3.6) and (3.7) to determine the first approximation for *γ_i_*, which is then substituted in Equation (3.10) to find the next approximation for *c_i_*, *etc*. The iterations are fast convergent: less than 20 iterations are sufficient to determine the values of *c_i_* and *γ_i_* with accuracy better than 14 significant digits.
2. From Equations (3.7) and (3.8) with *c_i_* = *c_i_*_m_, we determine *p*(Φ_m_), and then from Equations (3.9) and (3.11) we calculate Π_el_(Φ_m_).
3. For a given value of Φ_s_, from Equations (3.6) and (3.10) we determine the respective values of the concentrations and activity coefficients, *c_i_* = *c_i_*_s_ and *γ_i_* = *γ_i_*_s_, by iterations. The procedure is analogous to that in point 1 above.
4. With the same value of Φ_s_, Equation (3.16), is solved numerically (e.g., by the bisection method) to determine Γ_1_. Further, Γ_2_ is calculated from Equation (3.17).
5. From Equations (3.7) and (3.8) with *c_i_* = *c_i_*_s_, we determine *p*(Φ_s_), and then we solve Equation (3.15) numerically to determine Φ_s_.
6. The integral in Equation (3.13) is solved numerically. For this goal, at each given Φ from Equations (3.6) and (3.10) we determine the respective values of the concentrations and activity coefficients, *c_i_* and *γ_i_*, by iterations. The procedure is analogous to that in point 1 above. Next, from Equations (3.7) and (3.8) we calculate *p*(Φ_s_). As a result of integration, we determine *h*(Φ_m_).
7. The value of Φ_m_ is varied to obtain the dependences Π_el_ = Π_el_(Φ_m_) and *h* = *h*(Φ_m_).

### 3.3. Conventional DLVO Theory of Electrostatic Disjoining Pressure

For the conventional DLVO theory, one can find the dependences Π_el_ = Π_el_(Φ_m_) and *h* = *h*(Φ_m_) by using the same procedure (Section 3.2), but instead of Equations (3.8) and (3.9) the following simpler expressions have to be used to calculate the pressures:

(3.19) $$p(\Phi )=kT\sum_{i=1}^{n}c_{i\infty }\exp(-z_{i}\Phi )\;;\;p_{\infty }=kT\sum_{i=1}^{n}c_{i\infty }$$

In addition, the activity coefficients have to be expressed from the Debye–Hückel limiting law [5]:

(3.20) $$\ln\gamma_{i\infty }=-\frac{z_{i}^{2}}{2}L_{B}\kappa_{\infty },\;\kappa_{\infty }^{2}=8\pi \;L_{B}I\;$$

where *κ*_∞_ is the conventional Debye screening parameter; and *I* is the bulk ionic strength in terms of *c_i_*_∞_; see Equation (2.1). Note that Equation (3.20) is a special case of Equation (3.6) for *κr_i_* → 0 and *κ* → *κ*_∞_.

Thus, in the framework of the conventional DLVO theory the activity coefficients have to be set equal to the Debye–Hückel activity coefficient in Equation (3.20), *γ_i_* = *γ_i_*_m_ = *γ_i_*_s_ = *γ_i_*_∞_, which is constant (independent of *x*). The only equations, where the activity coefficients *γ*_1∞_ and *γ*_2∞_ appear, are the adsorption isotherms, Equations (3.16) and (3.17). Upon substituting *γ_i_* = *γ_i_*_∞_ in Equation (3.10), the activity coefficients disappear from this equation. Then, Equation (3.10) represents an explicit expression for the concentrations *c_i_* and iterations are not necessary at steps 1, 3, and 6 of the procedure in Section 3.2.

### 3.4. Calculation of the Total Disjoining Pressure

For the considered free foam films, the total disjoining pressure, Π, is the sum of Π_el_ and the van der Waals component of disjoining pressure, Π_vw_:

(3.21) $$\Pi =\Pi_{\text{el}}+\Pi_{\text{vw}}\;,\;\Pi_{\text{vw}}=-\frac{A_{H}}{6\pi {\left(h+d\right)}^{3}}$$

As before, *h* is the thickness of the aqueous core; and *d* accounts for the thickness of the two surfactant adsorption layers. In our calculations, we are using the Hamaker parameter *A*_H_ for water and *h*_w_ = *h* + *d* is the experimental “water thickness” of the film. We recall that the experiment yields *h*_w_ insofar as the intensity of light reflected from the film is determined, assuming that the film has a uniform refractive index equal to that of water; see Section 2.

The Hamaker parameter, *A*_H_, is calculated from the formula [39]:

(3.22) $$A_{H}\;=\frac{3}{4}\;kT\frac{{\left(\varepsilon_{i}-\varepsilon_{j}\right)}^{2}}{{\left(\varepsilon_{i}+\varepsilon_{j}\right)}^{2}}+\frac{3h_{P}\nu_{e}}{4\pi }\frac{{\left(n_{i}^{2}-n_{j}^{2}\right)}^{2}}{{\left(n_{i}^{2}+n_{j}^{2}\right)}^{3/2}}\int_{0}^{\infty }\frac{\left(1+2\tilde{h}\xi )\exp(\;-2\tilde{h}\xi \right)}{{\left(1+2\xi^{2}\right)}^{2}}d\xi$$

where the retardation effect is taken into account; *h*_P_ = 6.63 × 10^−34^ J·s is the Planck constant; ν_e_ ≈ 3.0 × 10^15^ Hz is the main electronic absorption frequency; *n_i_* and *n_j_* are the refractive indexes, respectively, of the outer and inner phase; *ε_i_* and *ε_j_* are the respective relative dielectric constants; the dimensionless thickness $\tilde{h}$ is defined by the expression $\tilde{h}=2\;\pi \nu_{e\;}h_{w}n_{j}\;{\left(n_{i}^{2}+n_{j}^{2}\right)}^{1/2}/c_{0}$; *c*_0_ = 3.0 × 10^8^ m/s is the speed of light in vacuum; and *ξ* is an integration variable.

## 4. Numerical Results and Discussion

### 4.1. Results for 1:1, 1:2, and 1:3 Electrolytes

For our system, *n_i_* = 1 and *ε_i_* = 1 (air); *n_j_* = 1.33 and *ε_j_* = 78.25 (water). *A*_H_ calculated from Equation (3.22) decreases from 3.0 × 10^−20^ J at *h*_w_ = 5 nm to 1.7 × 10^−20^ J at *h*_w_ = 20 nm because of the electromagnetic retardation effect. In the experimental range of film thicknesses, Π_wv_ is relatively small, but it is not negligible. For example, for the leftmost experimental point of the curve for Na_2_SO_4_ in Figure 2 we have *h*_w_ = 13.2 nm, Π = 7.10 kPa, and Π_el_ = 7.59 kPa, whereas |Π_wv_| = 0.49 kPa, *i.e.* 6.5% of Π_el_.

In the isotherms of SDS adsorption and Na^+^ binding, Equations (3.16) and (3.17), the following parameter values were used [30]: *K*_1_ = 100.1 (mM)^−1^; *K*_St,2_ = 6.529 × 10^−4^ (mM)^−1^; Γ_∞_^−1^ = 0.30 nm^2^ and 2*β*Γ_∞_/(*kT*) = 3.56. For water at 25 °C, the Bjerrum length is *L*_B_ = 0.716 nm.

For the hydrated ionic radii, *r_i_*, we used the values 0.36 nm for Na^+^ and 0.33 nm for Cl^−^ [3]; 0.31 nm for SO_4_^2^^−^ [29] and 0.36 nm for Citrate^3^^−^ [40,41]. For the surfactant anion with sulfate headgroup, we used the same *r_i_* as for SO_4_^2^^−^ [29]. Among all ions, the sodium counterions are present at a higher concentration in the EDL and, consequently, Π_el_ is the most sensitive to the radius of the Na^+^ ions. Conversely, the concentrations of the coins (repelled by the like charged film surfaces) are lower in the film, and Π_el_ is much less sensitive to the values of their radii.

The solid lines in Figure 2 are drawn by using the DLVO theory with ionic correlations (DLVO-IC), as described in Section 3.2. A single adjustable parameter was used, the effective water thickness of the surfactant adsorption layers, *d* in Equation (3.21); see also Figure 3. The three experimental isotherms in Figure 2 were simultaneously fitted with the DLVO-IC theory and *d* = 1.7 nm was obtained; the agreement between theory and experiment is excellent.

The dashed lines in Figure 2 represent the predictions of the conventional DLVO theory (see Section 3.3) with the same *d* = 1.7 nm in Equation (3.21). All constant geometrical and physical parameters used in the calculations with DLVO theory are the same as with DLVO-IC. In the case of a 1:1 electrolyte, NaCl, the prediction of DLVO theory coincides with that of DLVO-IC and with the experimental curve. However, in the cases of Na_2_SO_4_ and Na_3_Citrate the conventional DLVO theory predicts considerably greater values of Π in comparison with the experimental values and the predictions of DLVO-IC. This result confirms that the ionic correlation effects are really significant in the cases of divalent and trivalent coions.

It should be also noted that agreement between the conventional DLVO theory and experiment in the case of NaCl can be achieved only if we keep the activity coefficients *γ*_1∞_ and *γ*_2∞_ in the adsorption isotherms, Equations (3.16) and (3.17). For *I* = 31 mM, Equation (3.20) yields *γ*_1∞_ = *γ*_2∞_ = 0.845. If an approximation for an ideal solution (*γ_i_*_∞_ = 1) is used in the adsorption isotherms, the conventional DLVO theory predicts a Π(*h*_w_) isotherm, which is markedly above the experimental isotherm with NaCl in Figure 2.

**Figure 4:**
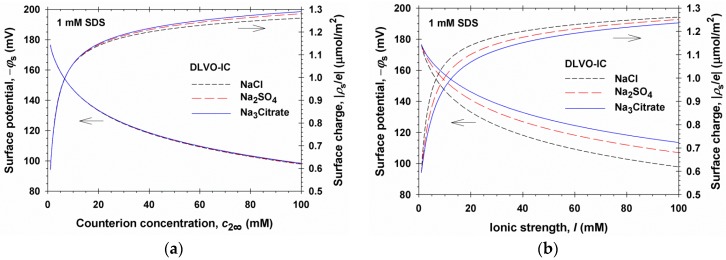
Plots of the surface potential, *ϕ*_s_, and surface charge, |*ρ*_s_/*e*| = Γ_1_ − Γ_2_, at a single air/water interface (*h* → ∞) (**a**) *vs.* the counterion (Na^+^) concentration, *c*_2∞_; (**b**) *vs.* the ionic strength, *I*: theoretical curves predicted by the DLVO-IC theory for a foam film formed from 1 mM SDS solution in the presence of NaCl, Na_2_SO_4_, and Na_3_Citrate.

Because all parameters in theory are known, we can calculate various properties of the liquid film in the framework of the DLVO-IC theory. Thus, in Figure 4a the dependences of the magnitudes of surface electric potential, |*ϕ*_s_|, and surface charge density, |*ρ*_s_/*e*| = Γ_1_ − Γ_2_, are plotted *vs.* the bulk concentration of counterions, *c*_2∞_, in the asymptotic case of large film thickness, *h* → ∞, *i.e.*, for a single interface. Three theoretical curves have been calculated, corresponding to the cases of added NaCl, Na_2_SO_4_, and Na_3_Citrate, *i.e.*, electrolytes with the same counterion, Na^+^, but with coions of different valence. It is remarkable that the three theoretical curves for |*ϕ*_s_| *vs.* *c*_2∞_, calculated for monovalent, divalent, and trivalent coions, practically coincide. This result, which is numerically very close to the prediction of DLVO, illustrates the known paradigm that the surface potential is determined by the counterion, and is insensitive to the valence of coions, which, in turn, has been used to predict the critical coagulation concentration (CCC) and to explain the Schulze–Hardy rule by the DLVO theory [1,2,3]. The decrease of |*ϕ*_s_| with the rise of *c*_2∞_ can be explained with the enhanced Debye screening. The increase of |*ρ*_s_/*e*| = Γ_1_− Γ_2_ with the rise of *c*_2∞_ could be attributed to the rise of surfactant (SDS) adsorption at the air/water interface, Γ_1_, upon the addition of electrolyte, owing to the suppression of the electrostatic repulsion between the surfactant ions at the interface. As seen in Figure 4a, a relatively weak effect of the coion valence on *ρ*_s_ is present at the higher values of *c*_2∞_.

Figure 4b shows the dependencies of surface potential and charge on the solution’s ionic strength, *I*, predicted by the DLVO-IC theory for the same 1:1, 1:2, and 1:3 electrolytes. In this case, the values of *ϕ*_s_ and *ρ*_s_ are different for the coions of different valence at the same ionic strength, *I*. This difference is almost completely due to the fact that at the same *I*, the counterion concentration, *c*_2∞_, is different in the 1:1, 1:2, and 1:3 electrolytes. For example, at 1 mM SDS and ionic strength *I* = 31 mM, we have *c*_2∞_ = 31, 21, and 16 mM in the solutions of NaCl, Na_2_SO_4_, and Na_3_Citrate, respectively. In view of Figure 4a different values of *c*_2∞_ correspond to different *ϕ*_s_ values.

**Figure 5:**
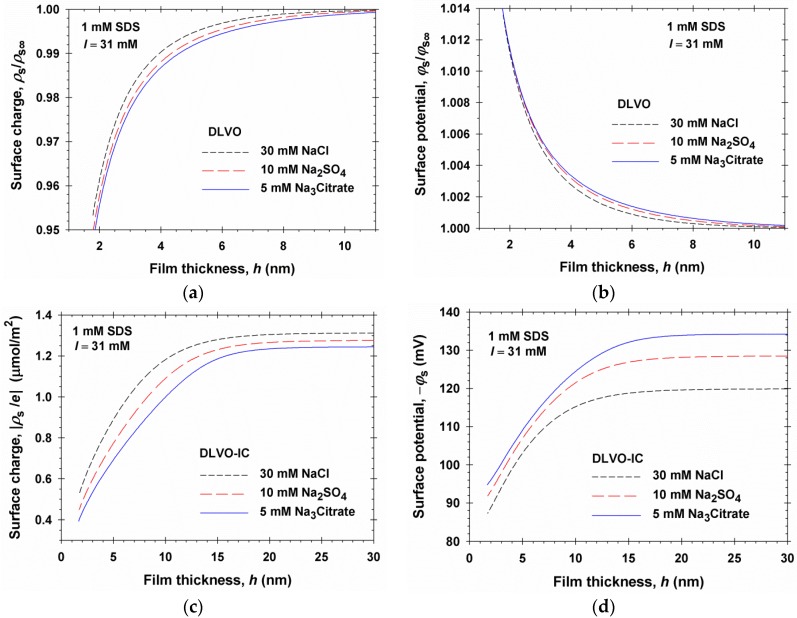
Comparison of the predictions of the DLVO and DLVO-IC theories with respect to the dependences of surface charge and potential, *ρ*_s_ and *ϕ*_s_, on the film thickness, *h*, for the same systems as in Figure 4. Predictions of DLVO theory for the dimensionless (**a**) surface charge, *ρ*_s_/*ρ*_s∞_; and (**b**) surface potential, *ϕ*_s_/*ϕ*_s∞_, scaled by their values at *h* → ∞; Predictions of DLVO-IC theory for (**c**) surface charge |*ρ*_s_/*e*| = Γ_1_ − Γ_2_; and (**d**) surface potential |*ϕ*_s_|.

In Figure 5, we compare the predictions of the DLVO and DLVO-IC theories with respect to the dependences of surface charge density and surface potential, *ρ*_s_ and *ϕ*_s_, on the film thickness, *h*. In the case of DLVO theory, Figure 5a,b, the results indicate the following: (i) *ρ*_s_ and *ϕ*_s_ are weakly sensitive to the valence of the coion. (ii) The dependencies of both surface charge and potential on *h* are rather weak: *ρ*_s_ varies with about 5%, whereas *ϕ*_s_ varies with only 1.4%. Hence, in this case the regime of charge regulation is closer to the regime of fixed surface potential. (iii) The magnitude of surface charge density, |*ρ*_s_|, decreases with the decrease of *h* (Figure 5a), which can be explained by the enhanced counterion binding in the thinner films; (iv) Conversely, the magnitude of the surface potential, |*ϕ*_s_|, increases with the decrease of *h* (Figure 5b), which is counterintuitive in view of the decrease in surface charge density |*ρ*_s_|.

As seen in Figure 5c,d, the predictions of the DLVO-IC theory essentially differ from those of the conventional DLVO theory: (i) The surface charge and potential are sensitive to the kind of the coion; (ii) the dependencies of both surface charge and potential on *h* are significant: |*ρ*_s_| increases about three times, and |*ϕ*_s_| increases from 90 mV up to 120–135 mV, depending on the coion; (iii) both surface charge, |*ρ*_s_|, and surface potential, |*ϕ*_s_|, decreases with the decrease of *h*, which can be explained with enhanced counterion binding in the thinner films. With respect to the behavior of |*ϕ*_s_| *vs.* *h*, the DLVO and DLVO-IC theories predict opposite tendencies; compare Figure 5b,d.

**Figure 6:**
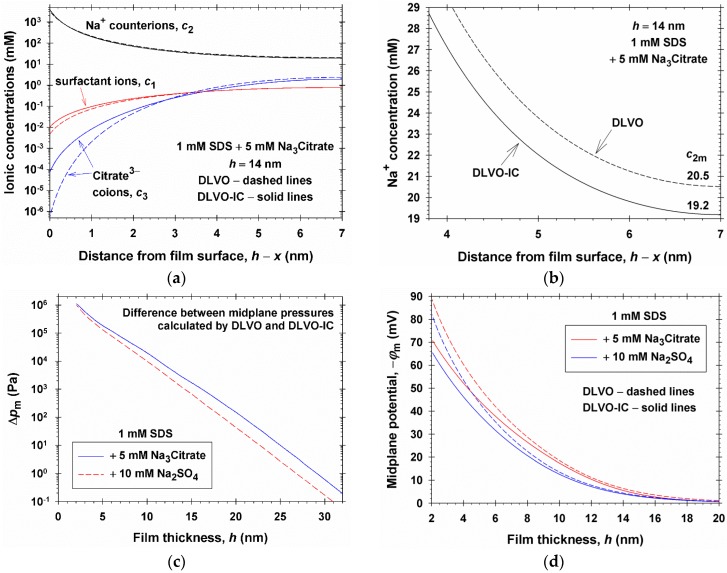
(**a**) Comparison of the predictions of DLVO and DLVO-IC theories for the variations of the concentrations of ionic species inside a film of thickness *h* = 14 nm formed from a solution of 1 mM SDS and 5 mM Na_3_Citrate; (**b**) The upper two curves in the previous plot shown in an enlarged scale; (**c**) Difference, Δ*p*_m_, between the midplane pressures calculated by the DLVO and DLVO-IC theories for films formed from solutions of 5 mM Na_3_Citrate and 10 mM Na_2_SO_4_ *vs.* *h*; (**d**) Comparison of the midplane potentials, *ϕ*_m_, calculated by means of the two theories, plotted *vs.* *h*.

To illustrate the differences between DLVO and DLVO-IC with respect to the ionic concentrations inside the film, in Figure 6 we have plotted the distributions of surfactant ions *c*_1_(*x*), counterions *c*_2_(*x*), and coions *c*_3_(*x*), *vs.* the distance to the film surface, *h*−*x*, predicted by the two theories for a film of thickness *h* = 14 nm formed from a solution of 1 mM SDS and 5 mM Na_3_Citrate. More significant relative differences between the two theories are seen for the surfactant and citrate coions, *c*_1_(*x*) and *c*_3_(*x*). Note, however, that the concentrations of coions in the film are much lower than those of the counterions, *c*_1_, *c*_3_ << *c*_2_, so that Π_el_ is dominated by the effect of counterion concentration, *c*_2_.

Our computations show that the difference between the Π(*h*) curves predicted by the DLVO and DLVO-IC theories for 1:2 and 1:3 electrolytes (Figure 2) is due mostly to the difference between the counterion concentrations, *c*_2m_, in the film midplane. To illustrate this fact, the two upper curves for *c*_2_ in Figure 6a (which seem to be almost coinciding) are plotted in magnified scale in Figure 6b. In this figure, *c*_2m_ is the value of *c*_2_ at *h* − *x* = 7 nm. The difference between the *c*_2m_ values predicted by the two models is Δ*c*_2m_ = 20.5 − 19.2 = 1.3 mM. In view of the first term in the right-hand side of Equation (3.8), this difference results in Δ*p* = *kT*Δ*c*_2m_ = 4.12 × 10^−21^ (J) × 1.3 (mol/m^3^) × 6.022 × 10^23^ (mol^−1^) = 3225 Pa pressure difference between the predictions of the two models. For comparison, the difference between the DLVO and DLVO-IC curves for Na_3_Citrate in Figure 2 at *h* = 14 nm (*h*_w_ = *h* + *d* = 15.7 nm) is ΔΠ = 3154 Pa.

In other words, Δ*p* gives the main contribution in ΔΠ. The relatively small difference between Δ*p* = 3225 Pa (estimated from the first term in Equation (3.8)) and the experimental ΔΠ = 3154 Pa, is due to the last term in Equation (3.8). The values of *p*_∞_ predicted by DLVO and DLVO-IC are very close; see Equations (3.9) and (3.11). In such a case, ΔΠ is practically equal to the difference, Δ*p*_m_, between the midplane pressures, *p*(Φ_m_), which is plotted *vs.* *h* in Figure 6c. As already mentioned, Δ*p*_m_ is dominated by the contribution of Δ*c*_2m_ that, in turns, is related to the difference between the midplane potentials *ϕ*_m_, which is illustrated in Figure 6d.

**Figure 7:**
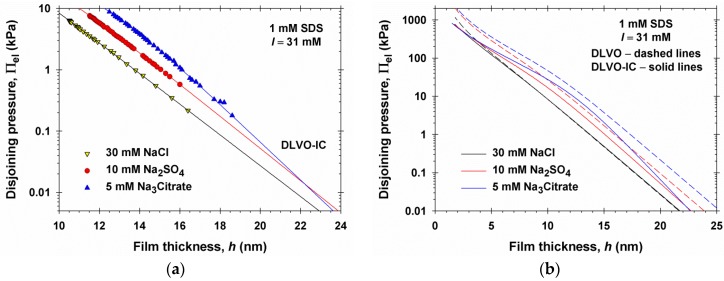
Asymptotic behavior of the electrostatic component of disjoining pressure, Π_el_, at large film thicknesses. (**a**) The experimental data from Figure 2 and theoretical curves predicted by DLVO-IC (the solid lines); (**b**) comparison of theoretical curves predicted by DLVO and DLVO-IC in wider range of Π_el_ and *h* values.

In Figure 7a, we have plotted the experimental data and DLVO-IC theoretical curves from Figure 2 as Π_el_ = Π − Π_vw_ *vs.* *h* in semi-logarithmic scale. Π_vw_ was calculated as explained in Section 3.4 with the same *d* = 1.7 nm, as above. We recall that the three experimental curves correspond to the same ionic strength, *I* = 31 mM, but to different coion valences, *z*_3_ = −1, −2 and −3. As seen in Figure 7a, at a large *h* value the experimental curves follow a linear dependence, which corresponds to an exponential asymptotic law:

(4.1) $$\Pi_{\text{el}}\propto \exp(-\kappa_{a}h)\mathrm{for}\mathrm{large}h$$

where *κ*_a_ is an asymptotic screening parameter. The values of *κ*_a_ determined from the computed curves at *h* → ∞ are given in Table 1, where they are compared with the value of the conventional Debye screening parameter, *κ*_∞_ = 0.580 nm. In general, *κ*_a_ ≥ *κ*_∞_, the difference being the greatest in the case of a 1:3 electrolyte, *viz.* 0.712 *vs.* 0.580 nm^−1^.

It is important to note that the experimental points obtained by the MJ cell do not belong to the asymptotic region of large *h*, where the slope of the curves in Figure 7a is characterized by *κ*_a_ in accordance with Equation (4.1). In fact, the experimental points correspond to smaller *h* values, where the curves for 1:1, 1:2, and 1:3 electrolytes are almost parallel (Figure 7a). To illustrate that, the points belonging to an experimental curve in Figure 7a (except the last one or two points with smaller Π, which are measured with lower accuracy) were fitted with linear regression. The fits are good (regression coefficients between 0.9964 and 0.9994); the slope of each regression yields an apparent screening parameter, *κ*_exp_, which is also given in Table 1. The values of *κ*_exp_ are close to *κ*_∞_. Hence, the slope of the semi-logarithmic plot of the experimental data cannot serve as an indicator for the significance of the effect of ionic correlations on the electrostatic surface force. The close values of *κ*_exp_ and *κ*_∞_ could be the reason for some authors to conclude that the force profiles in the presence of multivalent ions can be accurately described by the conventional DLVO (Poisson-Boltzmann) theory [12,13]. As demonstrated in Figure 2, the main effect of ionic correlations is the vertical shift of the experimental curves toward smaller Π values as compared with the DLVO prediction.

As demonstrated in Section 5, the asymptotic region characterized by slope *κ*_a_ in semi-logarithmic scale, see Equation (4.1), corresponds to the region with small midplane potential, Φ_m_ << 1. From this viewpoint, our experimental data (Figure 7a) correspond to a range of *h*, where the condition Φ_m_ << 1 is not satisfied (Figure 6d), and for this reason the local slope, *κ*_exp_, is different from *κ*_a_; see the data for Na_3_Citrate in Table 1.

Figure 7b compares the predictions of the DLVO and DLVO-IC for the same three systems, but in wider ranges of variation of *h* and Π_el_. The curves in Figure 7b indicate that the effect of ionic correlations affects Π_el_ at both short and long distances between the film surfaces. In the special case of a symmetric 1:1 electrolyte (NaCl), the differences between the predictions of the two theories are essential only at the smallest values of *h*, but for the asymmetric electrolytes these differences are essential for all *h* values. It is also remarkable that at the smallest *h* the effect of the different coion valences vanishes and the respective curves calculated by DLVO-IC almost coincide.

### 4.2. Results for a 2:2 Electrolyte

**Figure 8:**
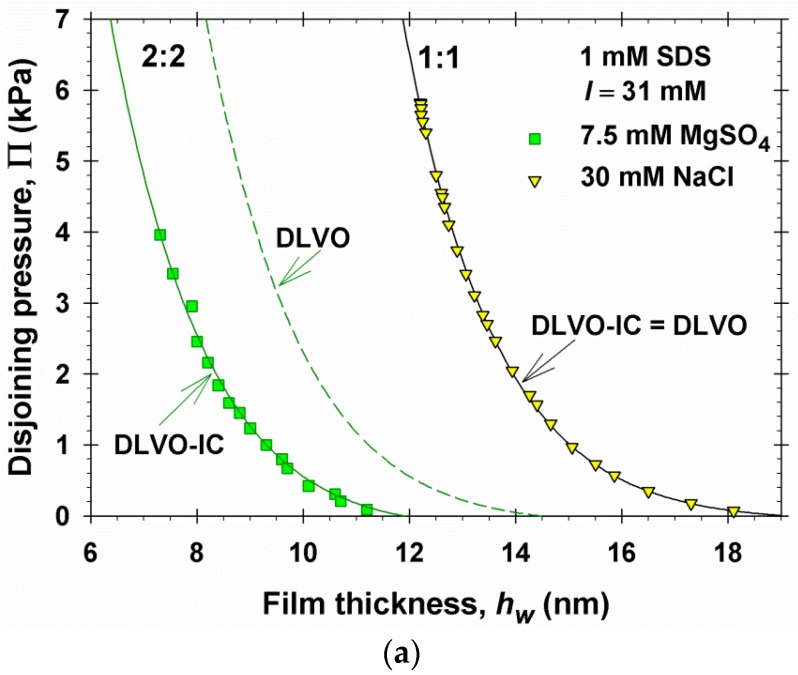

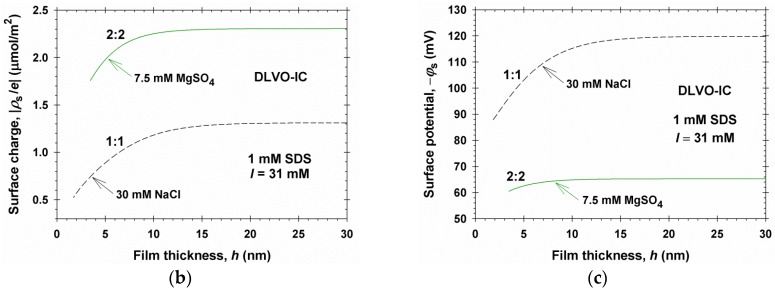
Comparison of results for 1:1 and 2:2 electrolytes at the same ionic strength, *I* = 31 mM. (**a**) Experimental data and theoretical curves predicted by DLVO-IC and DLVO theories; for a 1:1 electrolyte, the predictions of DLVO-IC and DLVO coincide; for a 2:2 electrolyte, the theoretical curves are drawn without using any adjustable parameters; (**b**) surface charge |*ρ*_s_/*e*|； and (**c**) surface potential |*ϕ*_s_| predicted by DLVO-IC.

In the case of a symmetric 1:1 electrolyte (*I* = 31 mM), no differences between DLVO and DLVO-IC have been observed; see Figure 2. It is interesting to verify whether there are differences between DLVO and DLVO-IC in the case of a symmetric 2:2 electrolyte. For this goal, we measured experimentally Π *vs.* *h*_w_ for foam films by using the MJ cell at the same ionic strength, *I* = 31 mM, which is due to 7.5 mM MgSO_4_, and 1 mM SDS; see Figure 8a. Because the radius of the hydrated Mg^2+^ ion and the Stern constant characterizing the binding of the Mg^2+^ ion to the surfactant headgroup are known (see below), the theoretical curves for a 2:2 electrolyte in Figure 8a predicted by the DLVO and DLVO-IC theories have been drawn without using any adjustable parameters. Details are following.

As seen from the experimental values of *h*_w_ (Figure 8a), in the presence of MgSO_4_ the equilibrium foam films are considerably thinner than in the presence of NaCl at the same ionic strength. Moreover, the maximal values of Π that can be achieved experimentally are not so high because a transition from primary (common black) film to secondary (Newton black) film takes place; see e.g., Figure 7.17 in reference [42].

Theoretically, in this case we are dealing with four ionic components, which are numbered as follows: 1—surfactant ion (dodecyl sulfate); 2—Na^+^ ion (from SDS); 3—SO_4_^2^^−^ anion and 4—Mg^2+^ cation (from MgSO_4_). All equations in Section 3 remain the same, except the surfactant adsorption isotherm, Equation (3.16) and the counterion binding isotherm, Equation (3.17). Equation (3.16) has to be replaced with the following equation [27]:

(4.2) $$\begin{array}{c} K_{1}\gamma_{1\infty }c_{1\infty }\exp(-\Phi_{s})=\frac{\Gamma_{1}}{\Gamma_{\infty }-\Gamma_{1}}\exp(\frac{\Gamma_{1}}{\Gamma_{\infty }-\Gamma_{1}}-\beta \frac{\Gamma_{1}}{\Gamma_{\infty }})\\ -K_{1}\gamma_{1\infty }c_{1\infty }K_{\text{St},2}\gamma_{2\infty }c_{2\infty }-K_{1}\gamma_{1\infty }c_{1\infty }K_{\text{St},4}\gamma_{4\infty }c_{4\infty }\exp(\Phi_{s}) \end{array}$$

where c_4∞_ and *γ*_4∞_ are the bulk concentration and activity coefficient of the Mg^2+^ ions and *K*_St,4_ is their Stern constant. Because both Na^+^ and Mg^2+^ counterions can adsorb in the Stern layer, Equation (3.17) has to be replaced with the following two Stern isotherms [27]:

(4.3) $$\frac{\Gamma_{2}}{\Gamma_{1}}=\frac{K_{\text{St},2}\gamma_{2\infty }c_{2\infty }\exp(\Phi_{s})}{1+K_{\text{St},2}\gamma_{2\infty }c_{2\infty }\exp(\Phi_{s})+K_{\text{St},4}\gamma_{4\infty }c_{4\infty }\exp(2\Phi_{s})}$$

(4.4) $$\frac{\Gamma_{4}}{\Gamma_{1}}=\frac{K_{\text{St},4}\gamma_{4\infty }c_{4\infty }\exp(2\Phi_{s})}{1+K_{\text{St},2}\gamma_{2\infty }c_{2\infty }\exp(\Phi_{s})+K_{\text{St},4}\gamma_{4\infty }c_{4\infty }\exp(2\Phi_{s})}$$

where Γ_4_ is the adsorption of Mg^2+^ ions. The Stern constant for Mg^2+^ ions, *K*_St,4_ = 1.92 × 10^−4^ (mM)^−1^, has been determined in reference [43] from data for the surface tension of SDS solutions in the presence of MgSO_4_. The radius of the Mg^2+^ ion is also known, *r*_4_ = 0.43 nm [3]. The values of all other physical parameters are the same as in Section 4.1.

The most important conclusions from Figure 8a are as follows. (i) It is remarkable that the DLVO-IC theoretical curve, drawn without using any adjustable parameters, is in excellent agreement with the experimental data for 2:2 electrolytes. (ii) The values of Π predicted by DLVO theory in the case of a 2:2 electrolyte are considerably greater than the experimental ones. (iii) The Π(*h*_w_) isotherm for a 2:2 electrolyte, as compared to that for a 1:1 electrolyte (at the same ionic strength), is shifted considerably to the left, which indicates stronger suppression of the electrostatic repulsion in the thin liquid films.

To understand the reasons for the last effect, in Figure 8b,c we have plotted the surface charge and surface potential as predicted by DLVO-IC. The surface charge is greater in the case of a 2:2 electrolyte (Figure 8a), because the binding constant of the Mg^2+^ ions, *K*_St,4_, is 3.4 times smaller than that of the Na^+^ ions, *K*_St,2_ (see above). This could be explained by the greater degree of hydration of the Mg^2+^ ions. In contrast, the surface potential, |*ϕ*_s_|, is about 2 times lower in the case of a 2:2 electrolyte (Figure 8b). This is due to the higher valence of the Mg^2+^ ions, which leads to a significant rise of their concentration in the subsurface layer, in accordance with the Boltzmann law, Equation (3.10). In other words, the more effective screening of the electrostatic interactions in the case of a 2:2 electrolyte is due to the increased concentration of divalent counterions in the diffuse part of the electric double layer, which also leads to enhancement of the ion-correlation effects, as evidenced by the difference between the DLVO and DLVO-IC disjoining pressure isotherms in Figure 8a.

## 5. Analytical Expression for the Asymptotic Screening Parameter

Here, we derive an analytical expression for the asymptotic screening parameter, *κ*_a_, which allows calculation of this parameter from the valences and radii of the ions present in the solution.

In terms of the dimensionless electric potential, Φ, the Poisson equation can be expressed in the form:

(5.1) $$\frac{d^{2}\Phi }{dx^{2}}=-\frac{e^{2}}{\varepsilon_{0}\varepsilon \;kT}\sum_{i=1}^{n}z_{i}(c_{i}-c_{i\infty })$$

where we assume that the bulk solution is electroneutral, *i.e.*, Σ*_i_z_i_c_i_*_∞_ = 0. At sufficiently large values of the film thickness, *h*, the potential near the film’s midplane is low and we could write:

(5.2) $$\exp(-z_{i}\Phi )\approx 1-z_{i}\Phi \;;\;c_{i}\approx c_{i\infty }(1+\varepsilon_{ci})\;,\;\gamma_{i}\approx \gamma_{i\infty }(1+\varepsilon_{\gamma i})$$

where *ε_ci_* and *ε_γi_* are small quantities. Substituting the quantities in Equation (5.2) into Equation (3.10) and keeping the leading terms in the power expansion, we obtain:

(5.3) $$\varepsilon_{ci}+\varepsilon_{\gamma i}=-z_{i}\Phi \;(\mathrm{linearized}\mathrm{Boltzmann}\mathrm{equation})$$

To simplify the further derivations, let us assume that all counterions can be characterized with a mean ionic radius, *r*_m_. For *r_i_* = *r*_m_ (*i* = 1, …, *n*), without any approximations Equation (3.6) acquires a much simpler form:

(5.4) $$\ln\gamma_{i}=-\frac{z_{i}^{2}L_{B}}{2}\frac{\kappa }{\left(1+\kappa \;r_{m}\right)}$$

Because of the general character of Equation (5.4), it holds also in the bulk of solution:

(5.5) $$\ln\gamma_{i\infty }=-\frac{z_{i}^{2}L_{B}}{2}\frac{\kappa_{\infty }}{\left(1+\kappa_{\infty }\;r_{m}\right)}$$

where *κ*_∞_ is the conventional Debye parameter; see Equation (3.20). Equation (5.5) represents a generalized form of the Debye-Hückel limiting law and has already been derived in a different way [44]. Substituting $\gamma_{i}\approx \gamma_{i\infty }(1+\varepsilon_{\gamma i})$ in Equation (5.4) and expanding in series for small (*κ* − *κ*_∞_), in view of Equation (5.5) we derive:

(5.6) $$\varepsilon_{\gamma i}=-\frac{z_{i}^{2}L_{B}}{2{\left(1+\kappa_{\infty }\;r_{m}\right)}^{2}}(\kappa -\kappa_{\infty })$$

Further, we substitute Equation (5.6) into Equation (5.3) and multiply by *c_i_*_∞_:

(5.7) $$c_{i}-c_{i\infty }=z_{i}^{2}c_{i\infty }\frac{L_{B}(\kappa -\kappa_{\infty })}{2{\left(1+\kappa_{\infty }r_{m}\right)}^{2}}-z_{i}c_{i\infty }\Phi$$

Substituting $c_{i}\approx c_{i\infty }(1+\varepsilon_{ci})$ in the definition of *κ*, Equation (3.7), and expanding in series for small values of *ε_ci_* and (*κ* − *κ*_∞_), we obtain:

(5.8) $$\kappa_{\infty }(\kappa -\kappa_{\infty })=2\pi L_{B}\sum_{i=1}^{n}z_{i}^{2}(c_{i}-c_{i\infty })$$

To find an expression for the last term in Equation (5.8), we multiply Equation (5.7) with *z_i_*^2^ and sum up:

(5.9) $$\sum_{i=1}^{n}z_{i}^{2}(c_{i}-c_{i\infty })=\frac{L_{B}(\kappa -\kappa_{\infty })}{2{\left(1+\kappa_{\infty }\;r_{m}\right)}^{2}}\sum_{i=1}^{n}z_{i}^{4}c_{i\infty }-\sum_{i=1}^{n}z_{i}^{3}C_{i}\Phi$$

The substitution of Equation (5.9) in the right-hand side of Equation (5.8) yields:

(5.10) $$\frac{L_{B}(\kappa -\kappa_{\infty })}{2{\left(1+\kappa_{\infty }r_{m}\right)}^{2}}[\frac{\kappa_{\infty }{\left(1+\kappa_{\infty }r_{m}\right)}^{2}}{\pi L_{B}^{2}}-\sum_{i=1}^{n}z_{i}^{4}c_{i\infty }]=-\Phi \sum_{i=1}^{n}z_{i}^{3}c_{i\infty }$$

Finally, the substitution of (*κ* − *κ*_∞_) from Equation (5.10) into Equation (5.7), followed by substitution of the obtained expression for (*c_i_* − *c_i_*_∞_) in Equation (5.1), yields:

(5.11) $$\frac{d^{2}\Phi }{dx^{2}}=\kappa_{a}^{2}\Phi$$

where the screening parameter *κ*_a_ is defined as follows:

(5.12) $$\frac{\kappa_{a}^{2}}{\kappa_{\infty }^{2}}=1+\frac{4\pi^{2}(L_{B}^{3}/\kappa_{\infty }^{3})(\sum_{i=1}^{n}z_{i}^{3}c_{i\infty }{)}^{2}}{{\left(1+\kappa_{\infty }r_{m}\right)}^{2}-(\pi L_{B}^{2}/\kappa_{\infty })\sum_{i=1}^{n}z_{i}^{4}c_{i\infty }}$$

In the case of EDL at a single interface, the solution of Equation (5.11) reads:

(5.13) $$\Phi_{1}(x)=\Phi_{a}\exp(-\kappa_{a}x)$$

where *x* is the distance to the interface; and Φ_a_ is a constant pre-exponential factor of the asymptotics of electric potential. Furthermore, setting *r_i_* = *r*_m_ (*i* = 1, …, *n*), as above, and applying analogous series expansion for small Φ in Equation (3.8), one can derive:

(5.14) $$\Pi_{\text{el}}=A\Phi_{m}^{2}$$

where *A* is a constant coefficient in this asymptotic expression for Π_el_. Finally, following the approach by Verwey and Overbeek [2], we apply the superposition approximation for low potentials in the film midplane, *viz.* Φ_m_ = 2Φ_1_(*h*/2), which in view of Equations (5.13) and (5.14) yields:

(5.15) $$\Pi_{\text{el}}=4A\Phi_{a}^{2}\exp(-\kappa_{a}h)$$

Equations (5.13) and (5.15) imply that if the ionic correlations are taken into account, the asymptotic decay length of both the potential Φ_1_ and disjoining pressure Π_el_ is *κ*_a_^−1^, where *κ*_a_ is given by Equation (5.12). In other words, *κ*_a_ is the generalized screening parameter that takes into account the effects of ionic correlations and finite ionic radius, *r*_m_. The form of Equation (5.12) leads to the following conclusions:

(i) The screening parameter *κ*_a_ depends not only on the sum $\sum_{i}z_{i}^{2}c_{i\infty }$ that enters the expression for the conventional Debye parameter *κ*_∞_, but also on the sums $\sum_{i}z_{i}^{3}c_{i\infty }$ and $\sum_{i}z_{i}^{4}c_{i\infty }$. This leads to a considerable effect of ions of higher valence, including coions*,* on the Debye screening.
(ii) Equation (5.12) shows that *κ*_a_ ≥ *κ*_∞_, which leads to a stronger screening and weaker electrostatic repulsion as a result of the ionic correlation effect: compare the DLVO-IC with the DLVO curves in Figure 7b.
(iii) The effect of ionic correlations is a long-range effect*—*it influences the long-range asymptotics of Π_el_ and can be significant when the film thickness is tens of nanometers, not only in films a few nanometers thick.
(iv) If all dissolved electrolytes are symmetric, then $\sum_{i}z_{i}^{3}c_{i\infty }$= 0 and Equation (5.12) yields *κ*_a_ = *κ*_∞_, *i.e.*, the ionic correlation effect on the asymptotic decay length disappears for symmetric electrolytes, supposedly the electric potential Φ_m_ in the film’s midplane is low. This fact is related to the coincidence of the predictions of DLVO and DLVO-IC theories for NaCl in Figure 2.
(v) The ionic radius, *r*_m_, essentially affects the value of the screening parameter *κ*_a_. If we formally set *r*_m_ = 0 in Equation (5.12), the calculated *κ*_a_ will be markedly different from the asymptotic slope of the exact numerical solution in Figure 7b.

To calculate the values of *κ*_a_ in Table 1 from Equation (5.12), we have set the mean ionic radius equal to that of the hydrated Na^+^ counterions, which are the most abundant in the film, *i.e.*, *r*_m_ = 0.36 nm.

## 6. Conclusions

In this article, we report experimental data for the disjoining pressure of foam films stabilized by anionic surfactant in the presence of 1:1, 1:2, and 1:3 electrolytes: NaCl, Na_2_SO_4_, and Na_3_Citrate. The disjoining pressure predicted by the DLVO theory coincides with the experimental data in the case of a 1:1 electrolyte, but it is considerably greater than the measured pressure in the cases of divalent and trivalent coions. To achieve agreement with the experiment, the DLVO theory is extended to account for the effects of ionic correlations and finite ionic radii. Original analytical expressions are derived for the local activity coefficient, electrostatic component of disjoining pressure, and asymptotic screening parameter; see Equations (3.6), (3.8), (3.11) and (5.12). With the same parameters of the counterion-binding and van-der-Waals-disjoining-pressure isotherms, as for a 1:1 electrolyte, the curves predicted by the extended theory are in perfect agreement with the experimental data for 1:2 and 1:3 electrolytes. The obtained formula for the screening parameter, *κ*_a_, exactly predicts the asymptotic decay length of disjoining pressure. In the investigated range of ionic strengths and surface charge densities, the effect of ionic correlations turns out to be essential if 1:2, 1:3 and 2:2 electrolytes are present in the solution.

In comparison with the DLVO theory, the effect of ionic correlations leads to more effective screening of the electrostatic interactions in the electric double layer, as evidenced by the lower values of electric potential in the middle of the film (Figure 6d). This results in a lower concentration of counterions in the film’s midplane (Figure 6b) and lower disjoining pressure, as experimentally observed (Figure 2). The effect of ionic correlations on disjoining pressure is a result of a fine balance of electrostatic and osmotic effects, plus the effects of counterion binding (adsorption), which can be accurately described only by using the respective consistent system of equations; see Section 3.1 and Section 3.2.

In the considered case, it is possible to formally fit the experimental data using the DLVO theory by adjusting an effective (empirical) surface potential for each separate experimental curve or by introducing different adjustable Stern constants for the different experimental curves. However, physically the Stern constant *K*_St,2_ must be the same for the three curves in Figure 2 insofar as the counterions are the same (Na^+^) and the interface is the same (dense adsorption layer of SDS); moreover, *K*_St,2_ is known from independent surface tension measurements [27,28,29,30]. In such a case, the experimental data cannot be quantitatively interpreted by the DLVO theory without taking into account the effect of ionic correlations.

For a symmetric 1:1 electrolyte, no effect of ionic correlations was observed. In contrast, for a symmetric 2:2 electrolyte a considerable ionic-correlation effect is present (Figure 8a). The fact that in the latter case the calculated disjoining-pressure isotherm coincides with the experimental curve without using any adjustable parameters is an additional argument in favor of the correctness of the DLVO-IC theory.

The extended theory shows that the ionic-correlation effects are essential in the whole range of film thicknesses, from nanometers to tens of nanometers, *i.e.*, it affects both the short-range and long-range surface forces. The effect of ionic radius, *r_i_*, is coupled with the effect of ionic correlations and it affects even the long-range asymptotics of disjoining pressure; see Equation (5.12). Although the pressure in the thin liquid film can be presented as a sum of terms related to the ionic concentrations and activity coefficients, see Equation (3.8), the disjoining pressure cannot be presented as a sum of electrostatic and ionic-correlation terms (e.g., Π_el_ + Π_cor_), because the ionic correlations affect not only the activity coefficients, but also the ionic concentrations.

The extended DLVO theory with ionic correlations (DLVO-IC) is applicable to both multivalent coions and counterions. One possible extension of the present study is its application to systems with other multivalent counterions. We hope that the data analysis by DLVO-IC could remove some discrepancies between theory and experiment observed in studies with liquid films from electrolyte solutions.

## Figures and Tables

**Table 1:** Comparison of values of the Debye parameter: the experimental slope, *κ*_exp_, from the data in Figure 7a and the theoretical asymptotic screening parameter, *κ*_a_, calculated from the asymptotic slope of the theoretical curves or from Equation (5.12).

System	*κ*_∞_ (nm^−^^1^) Debye Parameter	*κ*_exp_ (nm^−^^1^) Experimental Slope	*κ*_a_ (nm^−^^1^) Equation (5.12)
30 mM NaCl	0.580	0.57 ± 0.01	0.580
10 mM Na_2_SO_4_	0.580	0.59 ± 0.01	0.603
5 mM Na_3_Citrate	0.580	0.68 ± 0.01	0.712

**Figure A1:**
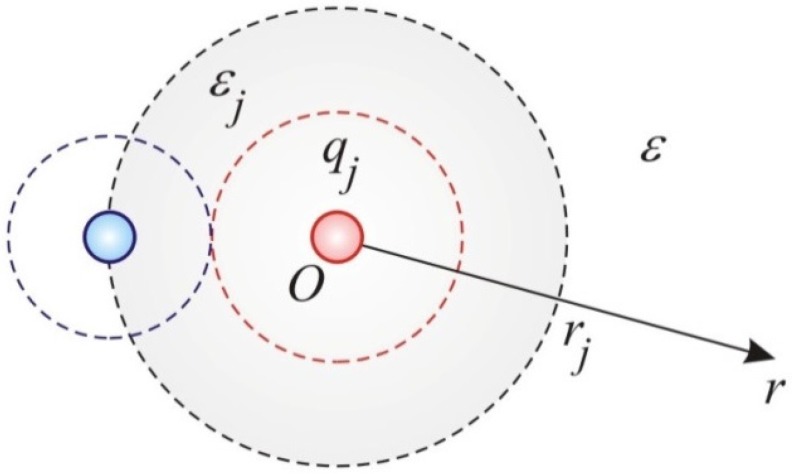
Sketch of two interacting ions in an electrolyte solution (details in the text).

## Appendix A. Derivation of the Expression for the Activity Coefficient

### A.1. Interaction Energy of the Individual Ions

Here, we give the derivation of Equation (3.6) for the activity coefficient *γ_i_*, which is the basis of the whole theory of the ion correlation effects developed in the present study. An electric double layer in a multicomponent electrolyte solution is considered and the effect of the finite size of the ions is taken into account. The approach by Debye and Hückel [5] is extended to a nonuniform phase (EDL) and Equation (3.6) for *γ_i_*, is obtained, which is an original result.

Let us consider the potential of the electric field, *ψ_j_*, created by a given ion in the EDL. The Poisson-Boltzmann equation can be written in the form:

(A1) $$\varepsilon_{0}\varepsilon \nabla^{2}(\varphi +\psi_{j})=-\sum_{i=1}^{n}q_{i}c_{i\infty }\exp[-\frac{q_{i}(\varphi +\psi_{j})}{kT}]$$

where *ϕ* is the mean field electrostatic potential created by the other ions in the EDL; *q*_1_, …, *q_n_* are ionic charges. The mean-field potential *ϕ* obeys the equation:

(A2) $$\varepsilon_{0}\varepsilon \nabla^{2}\varphi =-\sum_{i=1}^{n}q_{i}c_{i\infty }\exp[-\frac{q_{i}\varphi }{kT}]=-\sum_{i=1}^{n}q_{i}c_{i}$$

where *c_i_* are the mean local concentrations of the ionic species in the EDL.

The potential *ψ_j_* is assumed to be small enough, so that exp[−*q_i_ψ_j_*/(*kT*)] can be linearized. Then, for radial symmetry of the electric field around the selected ion, from Equations (A1) and (A2) we obtain:

(A3) $$\frac{1}{r^{2}}\frac{d}{dr}(r^{2}\frac{d\psi_{j}}{dr})=\kappa^{2}\psi_{j}$$

where *r* is the radial coordinate; and the Debye parameter *κ* is defined by Equation (3.7) in terms of the local concentrations, *c_i_*, with *q_i_* = *z_i_e*. Further, we use the Born model depicted in Figure A1, and present the electrostatic potential *ψ_j_* in the form:

(A4) $$\psi_{j}=\psi_{j,b}\frac{r_{j}}{r}\exp[-\kappa (r-r_{j})]\mathrm{for}r\geq r_{j}$$

(A5) $$\psi_{j}=\frac{z_{j}e}{4\pi \varepsilon_{0}\varepsilon_{j}r}+\psi_{j,\text{ind}}\mathrm{for}r\leq r_{j}$$

Equation (A4) expresses the solution of Equation (A3); *ψ_j_*_,b_ is the electrostatic potential at the boundary *r* = *r_j_* (see Figure A1). The form of Equation (A5) reflects the fact that for *r* ≤ *r_j_* there is no Debye screening; *ε_j_* is an effective dielectric constant for the region *r* ≤ *r_j_*; *ψ_j_*_,ind_ is a constant electric potential induced by the other ions. The constants *ψ_j_*_,b_ and *ψ_j_*_,ind_ in Equations (A4) and (A5) can be determined by the two boundary conditions at *r* = *r_j_*:

(A6) $${\psi_{j}|}_{r=r_{j}-0}={\psi_{j}|}_{r=r_{j}+0}\text{​}\mathrm{and}{\varepsilon_{j}\frac{d\psi_{j}}{dr}|}_{r=r_{j}-0}={\varepsilon \frac{d\psi_{j}}{dr}|}_{r=r_{j}+0}$$

The result for *ψ_j_*_,ind_ reads:

(A7) $$\psi_{j,\text{ind}}=-\frac{z_{j}e}{4\pi \varepsilon_{0}\varepsilon }\frac{\kappa }{1+\kappa r_{j}}-\frac{z_{j}e}{4\pi \varepsilon_{0}r_{j}}(\frac{1}{\varepsilon_{j}}-\frac{1}{\varepsilon })$$

The interaction energy of the selected ion with all other ions in the EDL can be estimated from the expression *u_j_* = *z_j_eψ_j_*_,ind_, which in view of Equation (A7) yields:

(A8) $$u_{j}=-\frac{z_{j}^{2}e^{2}}{4\pi \varepsilon_{0}\varepsilon }\frac{\kappa }{1+\kappa r_{j}}-\frac{z_{j}^{2}e^{2}}{4\pi \varepsilon_{0}r_{j}}(\frac{1}{\varepsilon_{j}}-\frac{1}{\varepsilon })$$

The first term in the right-hand side of Equation (A8) represents the Debye–Hückel interaction energy, whereas the last term is the Born energy.

### A.2. Free Energy and Electrochemical Potentials

The electrostatic interaction energy per unit volume of the EDL, which is due to the ionic correlations, is [5]:

(A9) $$u_{\text{int}}=\frac{1}{2}\sum_{j=1}^{n}c_{j}u_{j}$$

The combination of Equations (A8) and (A9) yields:

(A10) $$u_{\text{int}}=-\;\frac{e^{2}\kappa }{8\pi \varepsilon_{0}\varepsilon }\sum_{j=1}^{n}\frac{c_{j}z_{j}^{2}}{1+\kappa r_{j}}-\frac{e^{2}}{8\pi \varepsilon_{0}}\sum_{j=1}^{n}\frac{c_{j}z_{j}^{2}}{r_{j}}(\frac{1}{\varepsilon_{j}}-\frac{1}{\varepsilon })$$

The interaction energy, *u*_int_, and the respective free energy, *f*_int_, are related by the Gibbs-Helmholtz equation [6]:

(A11) $$\frac{u_{\text{int}}}{T^{2}}=-\;{\frac{\partial }{\partial T}(\frac{f_{\mathrm{int}}}{T})|}_{c_{1},c_{2},...,c_{n},\varphi }\Rightarrow \;f_{\mathrm{int}}=T\int_{T}^{\infty }\frac{u_{\text{int}}}{T^{2}}dT$$

Bearing in mind that *κ* = *κ*(*T*) (see Equation (3.7)), we integrate Equation (A10) in accordance with Equation (A11). The result reads:

(A12) $$f_{\text{int}}=\frac{e^{2}}{8\pi \varepsilon_{0}\varepsilon }\sum_{j=1}^{n}\frac{c_{j}z_{j}^{2}}{r_{j}}[\frac{2-\kappa r_{j}}{\kappa r_{j}}-\frac{2}{{\left(\kappa r_{j}\right)}^{2}}\ln(1+\kappa r_{j})]-\frac{e^{2}}{8\pi \varepsilon_{0}}\sum_{j=1}^{n}\frac{c_{j}z_{j}^{2}}{r_{j}}(\frac{1}{\varepsilon_{j}}-\frac{1}{\varepsilon })$$

The total free energy per unit volume can be presented in the form:

(A13) $$f=f_{n}+(\sum_{j=1}^{n}z_{j}ec_{j})\varphi +f_{\text{int}}$$

Here, *f*_n_ denotes non-electrostatic contributions to the free energy; the term with *ϕ* expresses the electrostatic energy in mean field approximation, and *f*_int_ is the interaction energy between the ions given by Equation (A12). Furthermore, the electrochemical potential of the *i*-th species can be calculated by differentiation:

(A14) $$\begin{array}{c} \mu_{i}={\left(\frac{\partial f}{\partial c_{i}}\right)}_{c_{j}\neq c_{i},\varphi }={\tilde{\mu }}_{i}^{o}+kT\ln c_{i}+z_{i}e\varphi -kT\frac{z_{i}^{2}L_{B}\kappa }{{\left(\kappa \;r_{i}\right)}^{3}}[\kappa \;r_{i}(\frac{\kappa \;r_{i}}{2}-1)+\ln(1+\kappa r_{i})]\\ -2\pi kT\frac{z_{i}^{2}L_{B}^{2}}{\kappa }\sum_{j=1}^{n}\frac{z_{j}^{2}c_{j}}{{\left(\kappa \;r_{j}\right)}^{2}}[\frac{2+\kappa \;r_{j}}{1+\kappa \;r_{j}}-\frac{2}{\kappa \;r_{j}}\ln(1+\kappa \;r_{j})]-\frac{e^{2}}{8\pi \varepsilon_{0}}\frac{z_{i}^{2}}{r_{i}}(\frac{1}{\varepsilon_{i}}-\frac{1}{\varepsilon }) \end{array}$$

The term ${\tilde{\mu }}_{i}^{o}+kT\ln c_{i}$ originates from the differentiation of *f*_n_; ${\tilde{\mu }}_{i}^{o}$ is a standard chemical potential. The electrochemical potential can be presented in the form:

(A15) $$\mu_{i}=\mu_{i}^{o}+kT\ln(\gamma_{i}c_{i})+z_{i}e\varphi$$

where *γ_i_* is the activity coefficient and

(A16) $$\mu_{i}^{o}\equiv {\tilde{\mu }}_{i}^{o}-\frac{e^{2}}{8\pi \varepsilon_{0}}\frac{z_{i}^{2}}{r_{i}}(\frac{1}{\varepsilon_{i}}-\frac{1}{\varepsilon })$$

The comparison of Equations (A14) and (A16) leads to Equation (3.6) for ln*γ_i_*.

It should be noted that the approach we used to the ionic correlation effects represents a two-step iteration procedure. At the first step, the Boltzmann equation in terms of concentrations is used in Equation (A1) to derive expressions for the electrochemical potential and activity coefficients. At the second step, the condition for uniform electrochemical potential leads to a new form of the Boltzmann equation, Equation (3.10), in terms of activities, which is further used to calculate the disjoining pressure. The results are applicable to ions of not too high a charge, for which the linearized Equation (A3) is valid.
